# Genetic Architecture of Migration-Related Traits in Rainbow and Steelhead Trout, *Oncorhynchus mykis*s

**DOI:** 10.1534/g3.112.003137

**Published:** 2012-09-01

**Authors:** Benjamin C. Hecht, Frank P. Thrower, Matthew C. Hale, Michael R. Miller, Krista M. Nichols

**Affiliations:** * Department of Biological Sciences, Purdue University, West Lafayette, Indiana 47907; † Alaska Fisheries Science Center, National Marine Fisheries Service, National Oceanic and Atmospheric Administration, Juneau, Alaska 99801; ‡ Institute of Molecular Biology, University of Oregon, Eugene, Oregon 97403; § Department of Forestry and Natural Resources, Purdue University, West Lafayette, Indiana 47907

**Keywords:** linkage mapping, migration, *Oncorhynchus mykiss*, QTL, RAD-tag sequencing, smoltification

## Abstract

Although migration plays a critical role in the evolution and diversification of species, relatively little is known of the genetic architecture underlying this life history in any species. Rainbow and steelhead trout (*Oncorhynchus mykiss*) naturally segregate for both resident and migratory life-history types, respectively, as do other members of the salmonid family of fishes. Using an experimental cross derived from wild resident rainbow and wild migratory steelhead trout from Southeast Alaska and high throughput restriction-site associated DNA (RAD) tag sequencing, we perform a quantitative trait locus (QTL) analysis to identify the number, position, and relative contribution of genetic effects on a suite of 27 physiological and morphological traits associated with the migratory life history in this species. In total, 37 QTL are localized to 19 unique QTL positions, explaining 4–13.63% of the variation for 19 of the 27 migration-related traits measured. Two chromosomal positions, one on chromosome Omy12 and the other on Omy14 each harbor 7 QTL for migration-related traits, suggesting that these regions could harbor master genetic controls for the migratory life-history tactic in this species. Another QTL region on Omy5 has been implicated in several studies of adaptive life histories within this species and could represent another important locus underlying the migratory life history. We also evaluate whether loci identified in this out-crossed QTL study colocalize to genomic positions previously identified for associations with migration-related traits in a doubled haploid mapping family.

Long-distance migrations undertaken by members of the animal kingdom are amid nature’s greatest spectacles. Migration is a universal theme among animals and plays a critical role in the health and balance of ecological systems (Wilcove and Wikelski 2008) and in the diversification and evolution of species (McDowall 2009). This phenomenon is triggered by environmental cues that begin a cascade of complex physiological, morphological, and behavioral changes, collectively called a “migratory syndrome” (Dingle 2006). Despite the overall importance of animal migrations to ecological systems and their rapid global decline (Wilcove 2008), we know relatively little about the underlying genetic architecture of this complex life history.

It is broadly understood that a significant proportion of the phenotypic variance in migratory traits is genetic (Dingle 1991; Liedvogel *et al.* 2011), but the individual genes, gene × gene, and gene × environment interactions involved in shaping these phenotypes are still widely unknown. Identifying genetic regions associated with migratory traits has proved difficult. Much of the challenge lies in the fact that most migratory species are not model systems, and they have few genetic and genomic resources available to them. Identifying and quantifying key migratory phenotypes can also be problematic (Liedvogel *et al.* 2011), making it difficult to design robust genetic mapping projects that can identify genomic regions associated with the traits.

Among the animals that undertake long-distance migrations, salmonids, members of the trout and salmon family of fishes, make ideal candidates for the study of the genetics of migration. Tremendous variation exists both within and between these species in the propensity, timing, distance, and duration of their marine migrations (Quinn and Myers 2004). Migratory salmonid fishes are born and reared in freshwater, migrate to the ocean as juveniles, and return to freshwater to spawn in what is called an “anadromous” life history. Alternatively, some individuals of these species will remain in freshwater habitat throughout their entire life cycle as “resident” life-history types (Quinn 2005).

To adapt to marine conditions, juvenile salmonids undergo a suite of physiological, biochemical, morphological, and behavioral changes within their natal freshwater habitat in a process called “smoltification” (Folmar and Dickhoff 1980; Hoar 1976). This process is cued by environmental changes, including increases in day length and water temperature (Clarke *et al.* 1978; Folmar and Dickhoff 1980; Hoar 1976). During this process, freshwater-adapted juvenile salmonids transform from dark-colored resident “parr” to silvery-colored “smolts,” which are physiologically and morphologically adapted to life in the ocean (Hoar 1976). The physiological and morphological changes that occur within migratory smolts do not occur to the same degree or extent in nonmigratory resident fish, allowing for the relative quantification of the smoltification process. Although the ecology and physiology of smoltification is well understood in salmonid fishes, relatively little is known of the genetic and molecular regulatory mechanisms underlying this process.

Nichols *et al.* (2008) first described the genetic architecture of physiological and morphological traits associated with migration *vs.* residency in *Oncorhynchus mykiss*, a Pacific salmonid species in which it has been shown that a heritable genetic component determines the migratory (steelhead trout) and resident (rainbow trout) life history (Johnsson *et al.* 1994; Thrower *et al.* 2004). Their study utilized two clonal lines of steelhead and rainbow trout, from Idaho and California, respectively, to identify several quantitative trait loci (QTL). Among those QTL identified were two promising regions, one on chromosome Omy5 and the other on chromosome Omy10, where several QTL colocalize. If the QTL on chromosome Omy10 truly harbor a master genetic switch for migration *vs.* residency in this species, as hypothesized by Nichols *et al.* (2008), we should expect that the same regions would be responsible for a significant proportion of the variation in migration-related traits from other crosses within this species. Alternatively, it is also possible that two populations exposed to different selection pressures could evolve independent genetic mechanisms leading to the same ultimate phenotype (Romano *et al.* 2010). In this scenario, different QTL could be detected when the same phenotypic trait is analyzed between crosses.

Here we aim to determine whether QTL for migration-related traits measured in a cross derived from wild steelhead and rainbow trout are the same as those found previously in domesticated clonal line crosses of steelhead and rainbow trout measured for similar migration-related traits (Nichols *et al.* 2008). QTL for similar traits localizing to the same genetic regions would indicate that conserved genetic mechanisms underlie components of the migratory syndrome within this species, whereas unique QTL between crosses might indicate the evolution of locally adapted genetic mechanisms. To more comprehensibly understand the genetic architecture of juvenile migration in rainbow trout and compare results with the prior study performed by Nichols *et al.* (2008), we conducted a QTL analysis of smoltification-related phenotypes in a cross derived from wild rainbow and steelhead trout from a system in southeast Alaska. Additionally, we took advantage of a high-throughput genotyping-by-sequencing method, wherein thousands of restriction site–associated DNA (RAD) tags are sequenced, aligned, and mined for SNPs.

## Materials and Methods

### Genetic crosses

Genetic analyses of juvenile smoltification-related traits were conducted using an out-crossed F_2_ breeding design derived from a cross of wild *O. mykiss* segregating for migratory life history (migratory *vs.* resident) from the Sashin Creek system on Baranof Island in southeast Alaska (Thrower *et al.* 2004). In total, 235 F_2_ offspring were raised in captivity from a single family in June of 2004. The breeding design, including a description of the P_1_ and F_1_ generations, are described in detail in the supplementary methods (see supporting information, File S1).

In June 2005 when F_2_ fish were approximately one year of age, fish were anesthetized using MS222 (Argent Laboratories, Redmond, WA) and tagged for individual identification using PIT tags (Biomark, Boise, ID). At that time, fin clips were collected and stored on Whatman paper for later isolation of DNA. Phenotypic measurements used for QTL analyses (described in detail below) were taken in June and September 2005, and then again in June 2006 at two years of age when life-history determination could be made.

### Phenotypes

To quantify the juvenile migratory phenotype in this species, we measured metrics of body size, condition, growth, morphology, skin reflectance, and osmoregulatory ability, all of which have been shown to vary between resident and migratory juveniles in this species. Body size and condition were measured at all three time points (June 2005, September 2005, and June 2006). Growth rates were measured in the consecutive intervals between these three time points. The remaining phenotypes were measured at age two in June 2006, the age and time at which smolts of this population and species are expected to out-migrate from Sashin Creek. Trait names used for QTL mapping are summarized in Table 1.

**Table 1:** Summary of the 27 smoltification-associated phenotypes measured for QTL analysis and the abbreviations assigned to them for reference throughout the text and in subsequent tables and figures

Phenotype	Abbreviation
Life-history classification [binary; smolts (1), residents (0)]	SMOLT
Fork length (June 2005)	Length605
Fork length (September 2005)	Length905
Fork length (June 2006)	Length606
Weight (June 2005)	Weight605
Weight (September 2005)	Weight905
Weight (June 2006)	Weight606
Body condition factor (June 2005)	Kfact605
Body condition factor (September 2005)	Kfact905
Body condition factor (June 2006)	Kfact606
Instantaneous growth in fork length (June 2005–September 2005)	IGRL1
Instantaneous growth in fork length (September 2005–June 2006)	IGRL2
Instantaneous growth in weight (June 2005–September 2005)	IGRW1
Instantaneous growth in weight (September 2005–June 2006)	IGRW2
Skin reflectance (average white pixel intensity)	AvgPix
Percentage weight lost during 24-hr seawater challenge	PWL
Blood plasma sodium concentration after 24-hr seawater challenge	BPNa
Centroid size	Centroid_Size
Relative warp 1–10	RelW1-RelW10

#### Life-history classification:

Individual life history (SMOLT) was scored as a binary trait at age two (June 2006). Smolts were identified qualitatively based on their overall body morphology and skin coloration, where smolting salmonids have more streamlined bodies with silvery reflective skin (Haner *et al.* 1995; Hoar 1976), and were assigned a value of 1. Resident, mature rainbow trout were identified by the positive expression of gametes (sperm or eggs) by gently applying pressure to their abdomen and assigned a value of 0. It is possible that those fish not expressing gametes and not exhibiting morphological features of migratory smolts could either undergo smoltification at a later age or become mature in freshwater, but to definitively identify resident rainbow trout and steelhead smolts, only these two life-history categories were scored. All fish not meeting these criteria were assigned missing values of NA for the life-history classification.

#### Body size, condition, and growth rate:

Fork length (distance from the tip of the snout to the fork of the caudal fin in millimeters) and body weight (in grams) was recorded for all fish in June 2005 (Length605 and Weight605), September 2005 (Length905 and Weight905), and June 2006 (Length606 and Weight606). Body condition, a measure of the relative contribution of body length on weight (Nash *et al.* 2006), was calculated with the formula (W/L^3^ × 100,000) for all sampling time points (Kfact605, Kfact905, and Kfact606). Smolting salmonids have a lower body condition when compared with nonsmolts (Folmar and Dickhoff 1980; Hoar 1976). Instantaneous growth rates in body length and body weight were calculated across two time periods, from June 2005 to September 2005 (IGRL/W1) and from September 2005 to June 2006 (IGRL/W2). The growth rate was calculated as [ln(L_2_) – ln(L_1_)]/[t_2_ – t_1_] × 100, where L_1_ and L_2_ are lengths or weights at the first (t_1_, in days) and second (t_2_, in days) time point in the interval being calculated. Smolting salmonids experience higher levels of growth in the spring of their second year compared with nonsmolts (Dickhoff *et al.* 1997; Folmar and Dickhoff 1980).

#### Body morphology:

**Figure 1:**
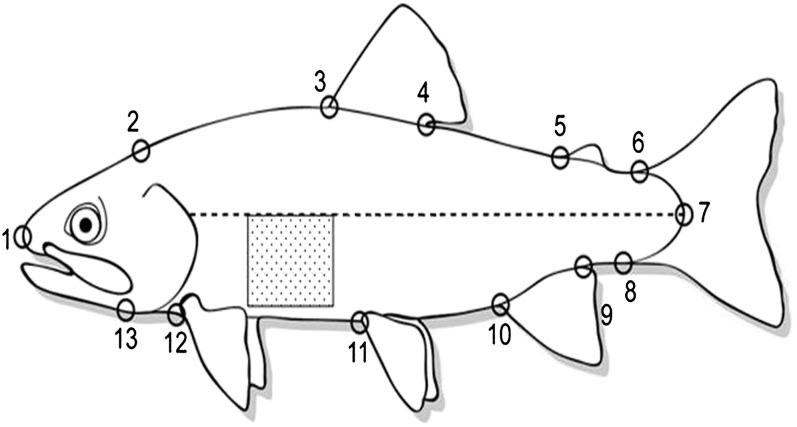
Digitized landmarks (1–13) for thin-plate spline analysis of body morphology and area measured for skin reflectance. Skin reflectance was quantified in the shaded region behind the pectoral fin, below the lateral line (dashed line) and before the insertion of the dorsal fin as the average white pixel intensity. Image used and modified with permission from Nichols *et al.* (2008).

Variation in body shape between smolts and nonsmolts has been well documented (Beeman *et al.* 1994; Beeman *et al.* 1995; Gorbman *et al.* 1982; Nichols *et al.* 2008). Smolts morph to a more streamlined and fusiform shape compared with deep-bodied resident rainbow parr. To quantify variation in body shape, we used a morphometric analysis of 13 landmarks along the left lateral side of the fish (Figure 1) as described by Nichols *et al.* (2008) and Varian and Nichols (2010). Digitized landmarks were analyzed for components of shape variation in a thin-plate spline analysis using *tpsRelW* (Rohlf 2008). From the 13 landmarks, 22 orthogonal components of shape variation called relative warps (RelW1-22) were estimated and are described below.

#### Skin reflectance:

As juvenile salmonids undergo the process of smoltification, metabolic byproducts guanine and hypoxanthine are deposited in the skin and scales, turning them from dark-colored parr to highly reflective, silver-colored smolts (Folmar and Dickhoff 1980). Skin reflectance in juvenile salmonids has been tightly linked to the deposition of guanine in the skin (Haner *et al.* 1995) and smoltification (Ando *et al.* 2005; Haner *et al.* 1995; Nichols *et al.* 2008). To quantify skin reflectance, the average white pixel intensity (AvgPix) of a defined region behind the pectoral fin, below the lateral line, and in front of the dorsal fin of each fish (Figure 1) was calculated from the same digital images used to identify landmarks for the morphometric analysis. This measure and the software used are further described in Nichols *et al.* (2008).

#### Seawater challenge:

In June 2006 after all other phenotypes had been collected, a subset of fish (n= 130) was exposed to a seawater challenge following the methods of Clarke (1982). Those fish with an increased capacity to osmoregulate should have the ability to expel excess Na^+^ ions from their blood stream and maintain their body weight in the saline environment. PIT tag and weight (in grams) of each individual was recorded at the beginning of the challenge before exposure. Fish were placed in a large vertical raceway in 30 ppt seawater at 9° for 24 hr. Fish were anesthetized following the challenge, and PIT-tag and weight (in grams) were again recorded to allow for the calculation of the percentage weight loss (PWL) during the seawater challenge. Percentage weight loss was calculated by: PWL = (W_AFTER_/W_BEFORE_) × 100, where W_BEFORE_ is body weight before 24-hr seawater challenge, and W_AFTER_ is body weight after the challenge. Change in body weight after exposure to seawater is a simple test of seawater readiness of smolts (Zaugg and Beckman 1990).

Immediately after the challenge, blood was drawn from the caudal vein and transferred to 1.5 mL centrifuge tubes. Plasma serum was separated from red blood cells by centrifugation at 4° for quantification of the blood plasma sodium concentration. Blood plasma was diluted to 2000× for quantification on an atomic absorption spectrophotometer. Blood plasma sodium (Na^+^) (BPNa) concentrations between 0.075 mM and 0.10 mM at 2000× were expected for 2-year-old juvenile steelhead smolts following 24-hr seawater challenge (McLeese *et al.* 1994). Potassium chloride (KCl) was added to all samples and standards to a final concentration of 2000 μg/mL to suppress the partial ionization of Na^+^ in the air-acetylene flame. Na^+^ flame emission was detected using a Shimadzu AA-6800 atomic absorption spectrophotometer (Shimadzu Scientific Instruments, Columbia, MD). A standard curve was generated from sodium standards, and this curve was used to estimate the blood plasma Na^+^ concentration for each sample.

### Genotyping

DNA was extracted from fin tissue using a phenol:chloroform procedure as outlined in Wasko *et al.* (2003). Genotypes were ascertained from grandparents (P_1_) and F_2_ (F_1_ parental DNA was not available) using microsatellite markers, a genetic sex marker, and SNP markers as described below.

#### Microsatellite markers:

In total, 165 microsatellite markers (see Table S1), selected to evenly span the genome based on previously generated microsatellite linkage maps in *O. mykiss* (Guyomard *et al.* 2006; Rexroad *et al.* 2008), were genotyped in the P_1_ grandparents and the F_2_. Details on PCR conditions for each marker type may be found in File S1.

#### Sex marker:

A genetic sex marker *OmyY1*, used herein as a proxy for phenotypic sex, was genotyped as described by Brunelli *et al.* (2008). The accuracy of this sex marker within the Sashin Creek population was verified in a mixed sample of 190 known-sex, mature adult fish from Sashin Lake and Sashin Creek (see File S1).

#### SNP markers:

SNP markers were scored for both putative candidate genes suspected to play a role in the migratory life history of salmonids and on the genome-wide level by next-generation sequencing of restriction site–associated DNA (RAD) tags (Baird *et al.* 2008; Miller *et al.* 2007). SNP markers in seven candidate genes were genotyped in all F_2_ progeny using the ABI SNaPshot Multiplex Kit (Life Technologies Corporation, Carlsbad, CA) on the ABI 3130 xL (Life Technologies Corporation, Carlsbad, CA) (Table S1). RAD tag sequencing was performed in a subset of 119 F_2_s, which had near complete phenotypic data. RAD libraries were prepared as described by Miller *et al.* (2012). Briefly, 500 ng of genomic DNA was digested with a restriction enzyme *Sbf*I (New England Biolabs, Ipswich, MA). Samples were ligated to adapters with barcodes and then pooled into libraries containing 10–25 (mean = 19) individuals per library. Libraries were PCR amplified and size selected to 400–600 bp before sequencing on an Illumina GAII-x or HiSeq 2000 (Illumina Inc., San Diego, CA) at a single read length of 80 (GAII-x) or 100 nucleotide bases (HiSeq 2000). A total of six libraries were constructed for the F_2_s. A single separate library was generated to sequence the four P_1_ grandparents and two doubled haploid samples OSU and SW described in further detail elsewhere (Miller *et al.* 2012; Nichols *et al.* 2007). Doubled haploid samples serve as completely homozygous genotype controls for identifying paralogous sequence variants (PSV), which cannot be easily distinguished in salmonids (Miller *et al.* 2012). All raw Illumina RAD-tag sequences generated for this study can be found in the NCBI Sequence Read Archive (http://www.ncbi.nlm.nih.gov/sra) under the project accession number SRA052219.

Genotyping and SNP discovery in RAD-tags was performed using perl scripts and a pipeline provided in Miller *et al.* (2012) and the alignment program Novoalign (Novocraft Technologies, Selangor, Malaysia). Details are outlined further in File S1.

### Statistics

SAS statistical software (SAS Institute Inc., Cary, NC) was used to carry out all statistical tests, unless otherwise noted. Phenotypes were evaluated for normality, homogeneity of variance, and outlier observations. Statistical tests for significance were made using a type I error rate of 0.05, unless otherwise indicated.

#### Sex:

Individuals with a female genotype using the sex marker *OmyY1* were assigned a phenotypic value of 0, and male genotypes were assigned a phenotypic value of 1. Analysis of variance (PROC GLM) was performed to test the hypotheses that phenotypic mean values were the same for both sexes (μ_m_ = μ_F_). Sex was used as an additive cofactor in QTL models for those phenotypes for which there was a significant sex effect.

#### Life history:

Analysis of variance (PROC GLM) was performed to test the hypotheses that phenotypic mean values were the same for both life-history classifications (μ_S_ = μ_R_). If sex was found to significantly contribute to the variation in a phenotype, that phenotype was conditioned on sex by adding sex as an effect in the model.

#### Correlation:

Correlation between traits used in the QTL analysis was estimated using Pearson’s correlations (PROC CORR) for normally distributed traits and Spearman’s correlations (PROC CORR) for correlation between traits and the binary SMOLT phenotype.

#### Thin-plate spline analysis:

Morphometric analysis of body shape variation was conducted using a thin-plate spline analysis calculated by the program tpsRelW (Rohlf 2008). An extended sample (n = 2053) of landmark data from full-sib and half-sib rainbow and steelhead trout families derived from Sashin Creek and Sashin Lake was used, so that body shape variation could be directly compared between studies of the heritability of smoltification-related traits (Hecht *et al.*, unpublished data) and the QTL analysis herein. These extended samples were raised in captivity and treated as outlined in File S1 and Thrower *et al.* (2004). Landmarks from fish were superimposed to calculate the generalized least-squares Procrustes average or consensus shape, while eliminating differences in body size (Rohlf and Slice 1990). Each sample was then analyzed for individual deformation from the consensus shape, and the variation is captured as partial warp scores (Rohlf and Bookstein 2003). Partial warp scores for all individuals were used in a principle-components analysis to condense partial warp scores into 22 orthogonal dimensions called relative warps (RelW1–RelW22). Of the 22 relative warps estimated, only 9 (RelW2–RelW10) were used as quantitative metrics for shape variation in downstream analyses (see *Results*).

#### Allometric growth:

Centroid size (Centroid_Size), which was measured as the squared root of the sum of squared distances of the 13 landmarks from the centroid, is a measure of the total body size of an individual. To account for variation in allometric growth for the relative warps, we tested the hypothesis that Centroid_Size had no effect on relative warp scores (RelW2–RelW10) using an analysis of variance (PROC GLM). Centroid_Size was used as a cofactor in QTL models of those relative warps that had a significant Centroid_Size effect. If both sex and Centroid_Size were found to be significant contributors to the variation in a relative warp, both were added as cofactors to models when testing for life history or QTL effects.

### Genetic linkage map and QTL analysis

Genetic linkage map construction was performed using a subset of 119 F_2_ individuals that had been genotyped for both microsatellite and RAD-tag SNP markers. Markers were filtered from the analysis if missing more than 30% data or if any doubled haploid sample was genotyped as a heterozygote (Miller *et al.* 2012). Markers were also removed for exhibiting extreme segregation distortion, determined by using chi-square tests and a Bonferroni-corrected *P* value (*P* = 0.05/659 = 0.000076). The genetic linkage map was constructed using the R statistical software package onemap (Margarido *et al.* 2007). The input data file containing all genotypes for the mapping panel for import into r/onemap is included as File S2. Additional information about the estimation of the genetic linkage map can be found in File S1. Quantitative trait loci analyses were performed using single, multiple, and two-dimensional QTL models in the R statistical package qtl (Broman *et al.* 2003). The input file containing all genotypes and phenotypes for import into r/qtl has been provided as File S3. Additional information about the estimation of the position, size, and effects of QTL can be found in File S1.

## Results

### Phenotypic traits

A single out-crossed F_2_ family of 223 individuals was measured for 27 physiological and morphological traits associated with smoltification including life-history classification at age two (SMOLT), genotypic sex (OmyY1), fork length at three time points (Length605, Length905, Length606), weight at three time points (Weight605, Weight905, Weight606), condition factor at three time points (Kfact605, Kfact905, Kfact606), instantaneous growth in fork length and weight across two time intervals (IGRL1, IGRW1, IGRL2, IGRW2), skin reflectance (AvgPix), percentage weight loss during seawater challenge (PWL), blood plasma sodium concentration after seawater challenge (BPNa), centroid size (Centroid_Size) and nine relative warp scores (RelW2–RelW10) summarizing variation in body morphology.

#### Life-history classification:

Of the 223 F_2_ progeny 76.23% (n = 170) were scored as steelhead smolts, 19.73% (n = 44) were scored as resident rainbow trout, and 4.04% (n = 9) exhibited intermediate phenotypes and were scored as missing data. Quantitative trait data are summarized below by life-history classification for all the traits used in QTL analyses to illustrate the differences between them.

#### Sex phenotype:

Of the 223 F_2_ progeny from the mapping family used for QTL analyses, 50.22% (n = 112) were genotypic females (as scored by the sex marker *OmyY1*), 48.43% (n = 108) were genotypic males, and 1.35% (n = 3) of individuals were undetermined, where the marker failed to amplify. Among those traits where sex explained a significant proportion of the variation (Table 2) was the life-history classification (SMOLT, *F* = 51.821, *P* = <0.0001), wherein 46.19% (n = 103) were female smolts, 1.35% (n = 3) were female residents, 28.69% (n = 64) were male smolts, 18.39% (n = 41) were male residents, and 5.38% (n = 12) were missing data for either sex or life history and could not be categorized. Of those that smolted, there was a significant difference between the proportion of females that smolted compared with the proportion of males that smolted, with 92% of the females smolting and only 59% of the males smolting. It is known that females have a higher tendency to smolt than males, partly due to an inherited difference in life-history tactics between the sexes (Dellefors and Faremo 1988; Jonsson *et al.* 1998). Sex also explained a significant proportion of the variation for Weight605 (*F* = 4.62, *P* = 0.0326), Weight905 (*F* = 9.12, *P* = 0.0028), Kfact905 (*F* = 35.83, *P* < 0.0001), Kfact606 (*F* = 37.44, *P* < 0.0001), IGRL2 (*F* = 9.42, *P* = 0.0024), IGRW2 (*F* = 5.59, *P* = 0.019), AvgPix (*F* = 239.53, *P* < 0.0001), PWL (*F* = 30.79, *P* < 0.0001), BPNa (*F* = 3.96, *P* = 0.0488), RelW2 (*F* = 62.76, *P* < 0.0001), RelW3 (*F* = 12.14, *P* = 0.0006), and RelW6 (*F* = 5.5, *P* = 0.02) (Table 2). For these traits, sex was used as an additive covariate in models of life-history effect and QTL.

**Table 2:** Sex effects on continuous phenotypic traits used for QTL analysis

Phenotype	Sex				
	Male (M)		Female (F)		*P*
	Mean	SE	Mean	SE	H_0_: μ_M_ =μ_F_
Length605	96.09259	1.02943	93.40000	1.02003	0.065
Length905	192.97170	1.64916	188.56482	1.63382	0.059
Lenght606	228.90566	2.18715	227.83178	2.17690	0.728
Weight605	10.35278	0.32887	9.35727	0.32586	0.033
Weight905	100.01132	2.59096	88.99815	2.56686	0.003
Weight606	141.76887	4.02048	132.22243	4.00165	0.094
Kfact605	1.12577	0.01139	1.10799	0.01129	0.269
Kfact905	1.36096	0.00832	1.29085	0.00824	<0.0001
Kfact606	1.15609	0.00870	1.08095	0.00866	<0.0001
IGRL1	0.66390	0.00569	0.67031	0.00564	0.425
IGRW1	2.16895	0.01777	2.15677	0.01760	0.627
IGRL2	0.06518	0.00161	0.07217	0.00161	0.002
IGRW2	0.13213	0.00447	0.14708	0.00447	0.019
AvgPix	65.97917	1.79152	71.18081	1.47876	0.027
PWL	7.72751	0.40328	4.37751	0.44933	<0.0001
BPNa	199.17181	2.45170	192.00356	2.63817	0.049
Centroid_Size	2140.62717	33.26014	2180.24590	33.09507	0.400
RelW2	−0.00080	0.00115	−0.01362	0.00114	<0.0001
RelW3	0.01177	0.00060	0.00881	0.00060	0.001
RelW4	0.00478	0.00079	0.00449	0.00079	0.795
RelW5	−0.00020	0.00082	0.00049	0.00081	0.552
RelW6	0.00227	0.00055	0.00409	0.00055	0.020
RelW7	0.00291	0.00052	0.00276	0.00052	0.836
RelW8	0.00227	0.00046	0.00195	0.00046	0.626
RelW9	0.00020	0.00047	0.00016	0.00047	0.949
RelW10	−0.00152	0.00054	−0.00105	0.00054	0.534

Sex: LS means ± SE (SE) of male (M) and female (F) effects on each trait, with *P* value for test of hypothesis μ_M_ = μ_F_.

#### Body size, condition factor, and growth:

Life-history classification only explained a significant proportion of the variation in fork length during the third time point in June 2006 (Length606, *F* = 15.92, *P* = <0.0001), with smolts having on average longer fork lengths than resident rainbows (Table 3). Life-history classification also explained a significant proportion of the variation in body weight during the second time point in September 2005 after accounting for sex (Weight905, *F* = 7.83, *P* = 0.0056), with resident rainbow trout having higher body weight than steelhead smolts (Table 3). For condition factor, life-history classification did not explain a significant proportion of the variation at the first time point; however, after accounting for sex, life-history classification explained a significant proportion of the variation for condition factor at the last two time points (Kfact905, *F* = 134.39, *P* = <0.0001; Kfact606, *F* = 208.94, *P* = <0.0001), with resident rainbow trout having the higher body condition at each time (Table 3). For instantaneous growth rate in length and weight calculated for the first interval between June 2005 and September 2005, life-history classification only explained a significant proportion of the variation in the growth in weight (IGRW1, *F* = 16.46, *P* = <0.0001), with resident rainbow trout having a higher overall growth rate in weight compared with steelhead smolts (Table 3). During the second time interval, between September 2005 and June 2006 and accounting for sex, life-history classification explained a significant proportion of the variation in growth rate in both length (IGRL2, *F* = 195.59, *P* = <0.0001) and weight (IGRW2, *F* = 120.52, *P* = <0.0001), with steelhead smolts exhibiting faster growth in both length and weight during this second time interval (Table 3).

**Table 3:** Life-history classification effects on continuous phenotypic traits used for QTL analysis

Phenotype	Life-history Classification				
	Resident Rainbow (RR)		Steelhead Smolt (SS)		*P*
	Mean	SE	Mean	SE	H_0_: μ_RR_ = μ_SS_
Length605	94.90909	1.59297	95.25000	0.81523	0.849
Length905	193.47727	2.48981	191.07143	1.27420	0.391
Lenght606	218.77273	3.08109	232.58333	1.57680	<0.0001
Weight605	**9.72290**	**0.55520**	**10.04140**	**0.26860**	**0.618**
Weight905	**106.12000**	**4.26820**	**92.40100**	**2.06510**	**0.006**
Weight606	137.09091	6.11324	139.24941	3.12855	0.754
Kfact605	1.14757	0.01643	1.11501	0.00841	0.079
Kfact905	**1.44220**	**0.01103**	**1.29540**	**0.00534**	**<0.0001**
Kfact606	**1.25300**	**0.01039**	**1.08040**	**0.00503**	**<0.0001**
IGRL1	0.67905	0.00876	0.66566	0.00448	0.175
IGRW1	2.26189	0.02634	2.14185	0.01348	<0.0001
IGRL2	**0.04539**	**0.00189**	**0.07573**	**0.00092**	**<0.0001**
IGRW2	**0.08184**	**0.00594**	**0.15670**	**0.00288**	**<0.0001**
AvgPix	**25.27810**	**2.91850**	**72.17890**	**0.70010**	**<0.0001**
PWL	**10.85140**	**0.34410**	**3.79260**	**0.21880**	**<0.0001**
BPNa	**213.32000**	**3.15090**	**187.58000**	**2.00820**	**<0.0001**
Centroid_Size	2111.85000	49.41540	2191.30000	25.94380	0.156
RelW2	**0.01278**	**0.00104**	**-0.01308**	**0.00052**	**<0.0001**
RelW3	**0.01461**	**0.00095**	**0.00911**	**0.00047**	**<0.0001**
RelW4	*0.00502*	*0.00117*	*0.00420*	*0.00061*	*0.540*
RelW5	−0.00131	0.00124	0.00054	0.00065	0.188
RelW6	***0.00225***	***0.00091***	***0.00353***	***0.00045***	***0.226***
RelW7	0.00239	0.00079	0.00279	0.00041	0.656
RelW8	0.00081	0.00069	0.00253	0.00036	0.029
RelW9	*0.00142*	*0.00071*	*-0.00007*	*0.00037*	*0.089*
RelW10	−0.00063	0.00083	−0.00146	0.00043	0.374

Life-history classification: LS means ± standard error (SE) of resident rainbow (RR) and steelhead smolt (SS) effects on each trait with *P* value for test of hypothesis μ_RR_ = μ_SS_. Bolded values are phenotypes conditioned on the effects of sex. Italicized values are phenotypes conditioned on the effects of centroid size.

#### Body morphology:

**Figure 2:**
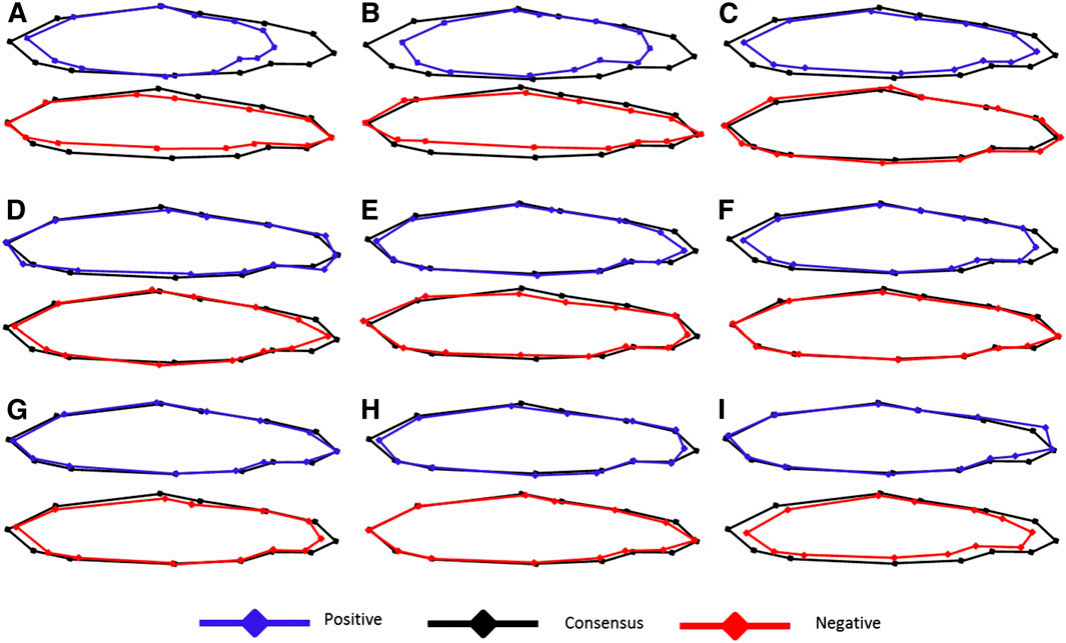
Relative warps from thin-plate spline analysis of body morphology. RelW2–RelW10 (A–I) explain collectively 54.55% of the variation in body shape. Extreme positive (blue) and negative (red) values of each relative warp are presented in comparison with the consensus shape (black).

Body morphology was analyzed in 204 F_2_ fish for QTL analysis, together with 1849 additional individuals used in another quantitative genetics study (Hecht *et al.*, unpublished data). Some individuals were missing from the analysis due to poor quality digital photographs. In total, 22 components of shape variation, called relative warps, were generated from the 13 landmarks. Of those 22 relative warps, the first 10 (RelW1–RelW10) explained 91.22% of the total shape variation, each of these explaining between 36.69% (RelW1) to 2.26% (RelW10) of the variation. Assessment of the shape variability defined by each of the relative warps (Figure 2) suggests that this analysis has successfully described variation in body morphology within this extended sample of two-year old rainbow and steelhead trout. When comparing the extreme positive and negative values for each relative warp with the consensus shape, much of the variation is captured as differences in (1) rear body length (landmarks 4, 5, 10, and 11); (2) caudal peduncle length and depth (landmarks 5–10); (3) dorsal-ventral body depth (landmarks 3, 4, 10, and 11); and (4) head and snout morphology (landmarks 1, 2, 12, and 13) (Figure 2). RelW1 explained the most variation in body shape and represented vertical bending of the body and extreme sagging of the tail, also noted in another study of body shape variation within fish (Albert *et al.* 2008). We believe this warp in body morphology is an artifact of anesthetization, and analyses revealed no QTL for this trait (data not shown); therefore, this warp was removed from any additional analyses. The percentage of body shape variation and a description of the shape variation explained by each relative warp are reported in Table S2. Mean relative warp differences between the sexes and the mean differences between the life-history classes are reported in Tables 2 and 3.

#### Skin reflectance:

Average white pixel intensity, calculated at age two in June 2006, was significantly different between life-history types. After accounting for sex, life-history classification explained a significant proportion of the variation in average white pixel intensity (AvgPix, *F* = 239.53, *P* < 0.0001), with steelhead smolts being more reflective and having higher values of average white pixel intensity than resident rainbow trout (Table 3).

#### Seawater challenge:

After accounting for sex, life-history classification explained a significant proportion of the variation in percentage weight loss following the seawater challenge test (PWL, *F* = 269.84, *P* < 0.0001), with resident rainbow trout losing a greater percentage of their body weight during the challenge compared with steelhead smolts (Table 3). Accounting also for sex, life-history classification explained a significant proportion of the variation in blood plasma sodium concentration (BPNa, *F* = 42.19, *P* < 0.0001), with resident rainbow trout having a higher concentration of sodium ion present in their blood plasma compared with steelhead smolts (Table 3).

### Correlation among traits

Of the 27 phenotypes measured, all but the tenth axis of morphometric shape variance (RelW10) was significantly (*P* ≤ 0.05) correlated with at least one other trait (see Table S3). The binary life-history trait (SMOLT) was correlated with all but nine of the traits. Percentage weight loss during seawater challenge (PWL) had the strongest negative correlation with the binary trait (*r* = −0.813, *P* < 0.0001), meaning resident rainbow trout lost more body weight during the challenge than steelhead smolts, although no QTL were detected for this trait. The strongest correlations existed between Length605 and Weight605 (*r* = 0.95, *P* < 0.0001) and Length905 and Weight905 (*r* = 0.96, *P* < 0.0001) and Length606 and Weight606 (*r* = 0.95, *P* < 0.0001). Two major QTL regions were identified in this study (outlined in detail below), Omy12 and Omy14, and each had seven QTL localize to the same region. For the QTL on Omy12 (SMOLT, Kfact905, Kfact606, IGRL2, IGRW2, Centroid_Size, RelW3) all of the pairwise trait correlations were significant except for those between Kfact905:Centroid_Size, Kfact606:Centroid_Size, and RelW3:Centroid_Size. For the QTL on Omy14 (IGRL1, IGRW1, Kfact905, Kfact606, RelW3, RelW4, and RelW9), all pairwise correlations were significant except for those between IGRL1:Kfact905, IGRL1:Kfact606, IGRL1:RelW3, IGRL1:RelW4, IGRL1:RelW9, IGRW1:RelW3, IGRW1:RelW4, and IGRW1:RelW9. Additional QTL that both colocalize to the same linkage group and show significant correlations include those on Omy4 (Centroid_Size:IGRW2, *r* = 0.23, *P* = 0.001), Omy18 (IGRL2:IGRW2, *r* = 0.91, *P* < 0.0001), and Omy27 (IGRW1:RelW2, *r* = 0.18, *P* = 0.0084).

### Genetic markers

Illumina sequencing of RAD-tags produced an average of 45 million quality-filtered reads per library across seven sequenced libraries, with an average of 2.3 million reads per individual. In total, 8790 polymorphic RAD-tag loci were identified within and between the two grandparents used for the index. Of the RAD loci discovered here, 3817 (43.4%) aligned perfectly to RAD-tag loci from another rainbow trout linkage map (Miller *et al.* 2012) and were named accordingly. Unique polymorphic RAD-tag loci from this study were named following the convention established in Miller *et al.* (2012), from R40650 to R45621 (see Table S4). By sequencing and genotyping two doubled haploid samples, we identified and removed 801 of the 8790 (9.1%) loci as putative PSVs. Tissue from F_1_ parents was not available; therefore, the F_1_ parents were not scored for any genetic markers. As a result, only those markers that could be unambiguously assigned a segregation pattern of inheritance between P_1_ grandparents and F_2_ progeny could be used for mapping. This resulted in the inclusion of a total of 609 microsatellite, SNP, and trait markers, which passed filtering criteria, in downstream linkage analyses.

### Genetic linkage map

Linkage mapping of 119 F_2_ offspring produced 31 major linkage groups, which falls within the haploid number of expected chromosomes in this species (Thorgaard 1983). Synteny among linkage groups between this linkage map and other rainbow trout linkage maps (Guyomard *et al.* 2006; Miller *et al.* 2012; Palti *et al.* 2011; Phillips *et al.* 2006; Rexroad *et al.* 2008) was established through commonly mapped syntenic microsatellite markers. Linkage groups were named and assigned chromosome numbers following the cytogenetic map of Phillips *et al.* (2006). Two rainbow trout chromosomes, Omy5 and Omy20, were represented by two linkage groups each in this cross and were named Omy5a/b and Omy20a/b, respectively. In total, 587 markers were mapped including, 164 microsatellite, 414 RAD-tag SNP, 7 candidate gene SNPs, 1 sex marker, and the binary trait SMOLT. Of the shared 3817 RAD-tag loci between this study and Miller *et al.* (2012), 58 were mapped in both, all of which were assigned to the same linkage group in both studies. The linkage map spanned a sex-averaged total of 4079 cM, with an average intermarker distance of 7.5 cM and a median intermarker distance of 6.6 cM. Twenty-two markers could not be joined to any of the major linkage groups at a LOD ≥ 5.0 and were, therefore, discarded. Life-history classification (SMOLT) was mapped as a dominant marker to chromosome OmySex to position 143 cM. It is not unexpected for this trait to map to the sex chromosome given the significant effect of sex on this life-history trait (Table 2); however, this marker was removed from the genetic linkage map to allow for the unbiased exploration of QTL. The relative map position and chromosome and linkage group assignments for all markers are given in Table S5.

### QTL analyses

**Figure 3:**
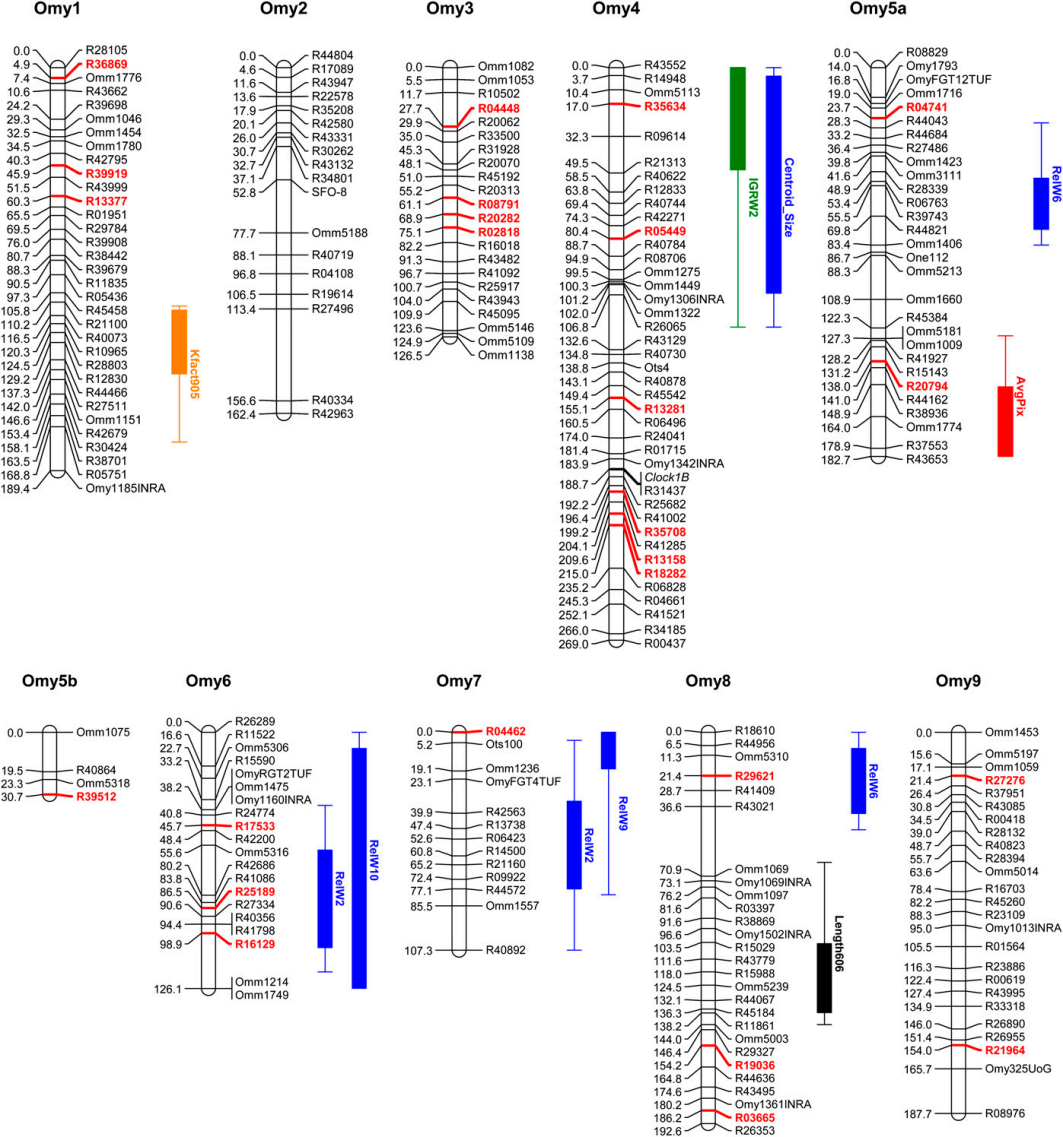

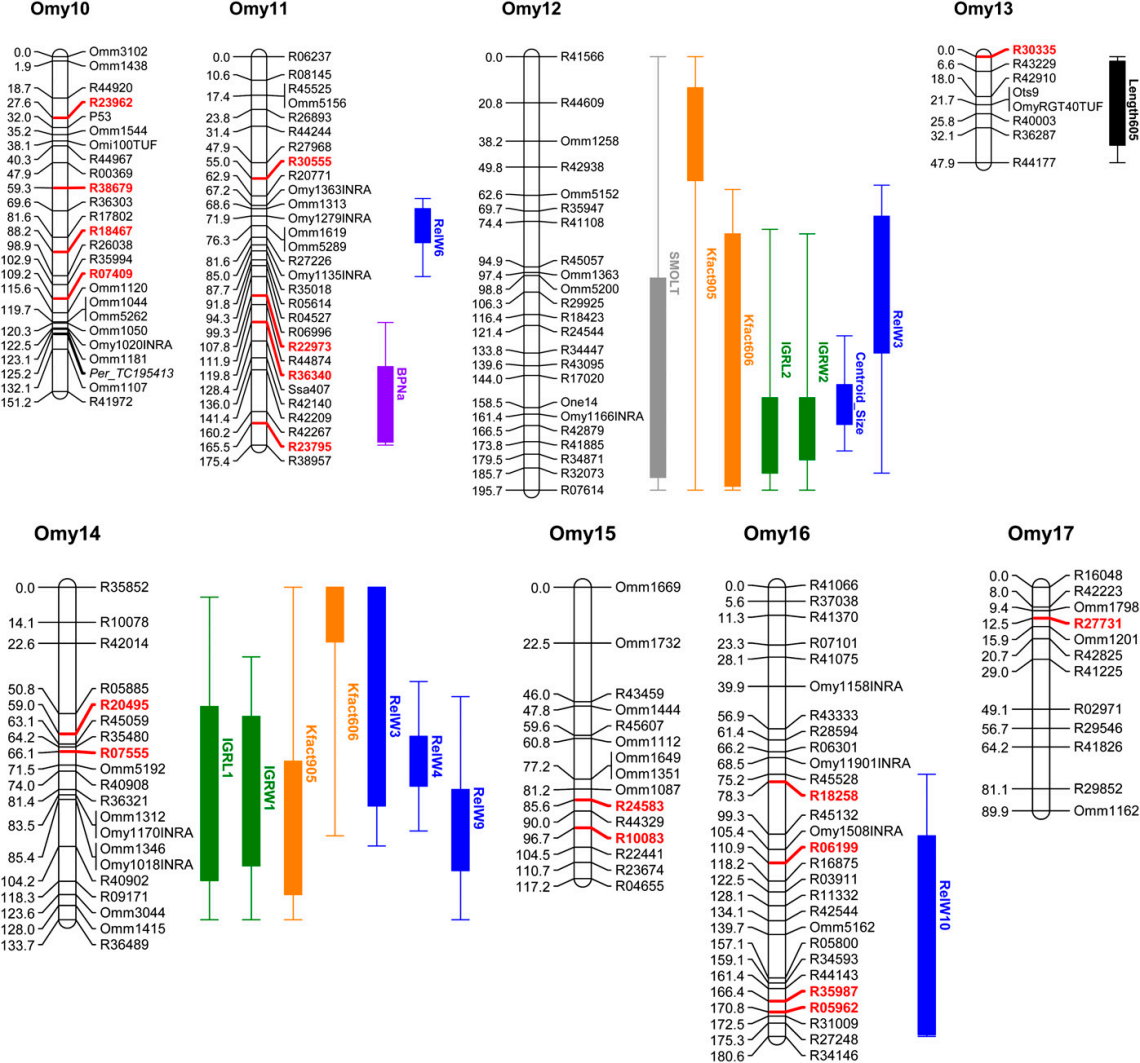

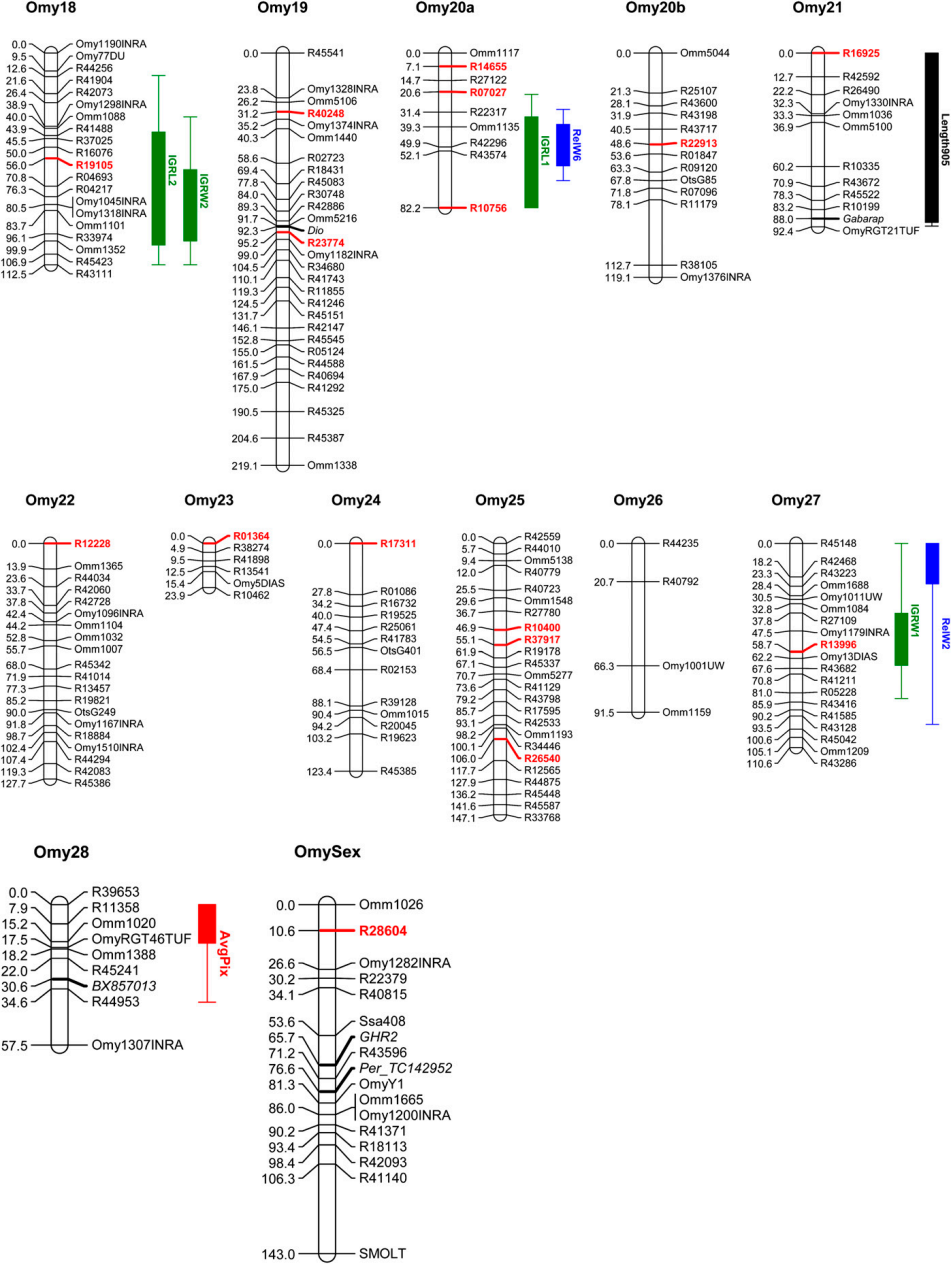
Genetic linkage map with linkage group assignment determined using syntenic markers with previously published rainbow trout maps. Loci in bold red font are RAD-tag SNP markers also mapped in Miller *et al.* (2012). Note marker SMOLT on OmySex was removed before QTL analysis. QTL for smoltification-related traits are shown as 2-LOD support intervals. Trait abbreviations are defined within the text or in Table 1.

Using multiple QTL models, 37 QTL explaining 4.01–13.63% of the variation were identified for 19 of the 27 smoltification-related traits and were classified as being significant at the genome- or chromosome-wide level, with no QTL reported if significance fell below a chromosome-wide 95% level. The 37 identified QTL map to 19 unique QTL positions, with two regions, Omy12 and Omy14, harboring 7 QTL each. QTL were not identified for Weight605, Weight905, Weight606, Kfact605, PWL, RelW5, RelW7, or RelW8. For all single traits that exhibited multiple QTL (Kfact905, IGRL1, IGRW1, Kfact606, IGRL2, IGRW2, AvgPix, Centroid_Size, RelW2, RelW3, RelW6, RelW9, and RelW10), two-dimensional QTL models were fit to test for epistatic interactions, although none of these interactions were significant above the genome-wide 90% threshold. Bonferroni correction for the *P* value of the 27 single traits tested is α = 0.05/27 or 0.0019, though this may not be an appropriate correction as not all of these 27 traits are strictly independent. Additionally the interpretation of *P* values in a multiple QTL framework requires caution, as the *P* values are pointwise estimates for models based on genome-wide searches (Broman and Sen 2009). Of the 37 QTL, 12 had *P* values greater than 0.0019 and should be interpreted with caution. QTL, their chromosome position, and percentage variation explained are detailed in Table 4 and illustrated along with the linkage map in Figure 3.

**Table 4:** Description of QTL identified

Phenotype	Chromosome	Position (cM)	QTL Peak	LOD	PVE	p(F)	Model Cofactor		Significance Threshold				
			Marker				Sex	Centroid_Size	GW99	GW95	GW90	CW99	CW95
SMOLT	Omy12	179.5	R34871	3.19	5.348	0.0021	x						x
Length605	Omy13	21.7	OmyRGT40TUF	3.58	7.18	0.001						x	x
Length905	Omy21	62	R10335	3.26	6.68	0.002						x	x
Length606	Omy8	132.1	R44067	3.31	6.81	0.0018						x	x
Kfact905	Omy1	129.2	R12830	4.36	6.81	0.00026	x			x	x	x	x
Kfact905	Omy12	32	Omm1258	3.42	5.29	0.0018	x					x	x
Kfact905	Omy14	110	R40902	3.77	5.85	0.0009	x					x	x
KFact606	Omy12	94	R45057	4.17	7.9	0.00033	x				x	x	x
KFact606	Omy14	2	R35852	3.47	6.5	0.0014	x					x	x
IGRL1	Omy14	62	R43574	2.99	5.6	0.0039							x
IGRL1	Omy20a	62	R43574	4.25	8.08	0.00028				x	x	x	x
IGRW1	Omy14	83.5	Omm1312	4.05	7.73	0.00042					x	x	x
IGRW1	Omy27	50	Omy1179INRA	3.28	6.2	0.0022						x	x
IGRL2	Omy12	174	R41885	3.73	6.85	0.00087	x					x	x
IGRL2	Omy18	80.5	Omy1045INRA	3.13	5.7	0.0031	x						x
IGRW2	Omy12	173.8	R41885	2.988	5.38	0.0043	x						x
IGRW2	Omy18	80	Omy1318INRA	3.336	6.03	0.0021	x					x	x
IGRW2	Omy4	38	R09614	3.89	7.08	0.00067	x				x	x	x
AvgPix	Omy28	10	R11358	5.45	13.63	0.000024	x			x	x	x	x
AvgPix	Omy5a	174	R37553	4.333	10.65	0.00026	x				x	x	x
BPNa	Omy11	150	R42209	3.48	11.81	0.0015	x						x
Centroid_Size	Omy12	160	Omy1166INRA	4.91	9.97	0.000068			x	x	x	x	x
Centroid_Size	Omy4	16	R35634	3.76	7.53	0.00079					x	x	x
RelW2	Omy27	12	R42468	4.05	5.92	0.00051	x				x	x	x
RelW2	Omy6	86	R25189	3.88	5.65	0.00073	x				x	x	x
RelW2	Omy7	60.8	R14500	2.79	4.01	0.0068	x						x
RelW3	Omy12	88	R45057	4.66	9.5	0.00012	x			x	x	x	x
RelW3	Omy14	63.1	R45059	3.3	6.6	0.0021	x					x	x
RelW4	Omy14	74	R40908	3.14	6.53	0.0027		x				x	x
RelW6	Omy11	74	Omy1279INRA	5.67	9.16	0.00002	x	x	x	x	x	x	x
RelW6	Omy20a	53.6	R01847	4.83	7.72	0.00011	x	x		x	x	x	x
RelW6	Omy5a	66	R44821	4.82	7.7	0.00011	x	x		x	x	x	x
RelW6	Omy8	28	R41409	5.65	9.11	0.00002	x	x	x	x	x	x	x
RelW9	Omy14	102	R40902	3.37	6.5	0.0019		x					x
RelW9	Omy7	10	Ots100	4.34	8.5	0.00025		x		x	x	x	x
RelW10	Omy16	134	R42544	3.04	6.23	0.0036							x
RelW10	Omy6	55.6	Omm5316	3.06	6.27	0.0034						x	x

Identification includes phenotype; chromosome; position (position of qtl peak on chromosome); QTL peak marker; LOD (log10 likelihood ratio for presence of QTL); PVE (percentage variation explained in phenotype by QTL); p(F) (*P* value of the F-statistic); model cofactor (indication of whether Sex or Centroid_Size was used as an additive cofactor in QTL model); and significance threshold at the GW (genome-wide) or CW (chromsome-wide) 99, 95, or 90% level.

## Discussion

We have performed a QTL analysis in a segregating F_2_ cross derived from wild resident rainbow and migratory steelhead trout and identified several genetic regions associated with both single and multiple physiological and morphological indices of migration. Our results combined with those from Nichols *et al.* (2008) are the only studies we know of which engage a QTL analysis of multiple physiological and morphological indices of migration in segregating crosses of migratory and resident life-history types of any species. The QTL that our combined studies have identified could lead to the discovery of candidate genes associated with the smoltification process and ultimately the decision to migrate or remain resident within this species. It is recognized that similar migratory syndromes exist across taxa (Dingle 2006), and although migration has evolved across taxa independently numerous times, common themes may represent parallel or convergent responses to the environmental demands of migratory life histories (Piersma *et al.* 2005; Ramenofsky and Wingfield 2007). Therefore, some of the key genetic components underlying migration in one species may be candidate in others.

Using a cross derived from domesticated clonal lines of rainbow and steelhead trout, Nichols *et al.* (2008) found a number of genetic regions associated with smoltification-related traits and two regions (Omy5 and Omy10) showing the joint localization of multiple QTL. And while QTL mapping can be a powerful statistical tool for identifying genetic regions of complex trait association (Doerge 2002), QTL analyses performed within a single cross may yield false-positive QTL. It is, therefore, recommended to repeat QTL analysis using different lines to validate QTL and identify QTL previously undetected (Falconer and Mackay 1996). One of our primary aims was to perform a QTL analysis in a different cross and to compare the QTL from this cross with those detected in Nichols *et al.* (2008) to determine whether the same genetic regions were found to be associated with similar smoltification-related traits from divergent populations of migratory steelhead trout. In total, five QTL regions contributing to smoltification-related traits are shared between the two studies. Those regions include QTL on chromosomes Omy5(a), 8, 12, 14, and 16. Here we discuss only those on chromosomes Omy5(a), 12 and 14, as the QTL shared between the crosses on Omy8 and Omy16 are for components of morphology, including RelW6 and RelW10 (in this study), which were not found to be significantly different between the two life-history types in this cross.

One region in common between studies on chromosome Omy5(a) is of particular interest, as this region is associated with multiple life-history traits and shows signatures of natural selection in natural populations. Nichols *et al.* (2008) identified QTL for growth rate in body length and weight, body morphology, and two joint QTL (where multiple smoltification-related traits were simultaneously analyzed for QTL) on this chromosome. In our study, we identified a QTL for body morphology (RelW6) and a QTL for skin reflectance (AvgPix), which is tightly correlated with the smoltification process, seawater adaptability, and the overall migratory life history (Ando *et al.* 2005; Folmar and Dickhoff 1980; Haner *et al.* 1995). QTL on this chromosome have been implicated in several studies as being significant contributors to life-history variation within rainbow trout for traits, such as smoltification and migration (Nichols *et al.* 2008), embryonic development rate (Miller *et al.* 2012; Nichols *et al.* 2007; Robison *et al.* 2001), growth (Wringe *et al.* 2010), early maturation (Haidle *et al.* 2008), and timing of maturation (O’Malley *et al.* 2003). In a genome-wide screen for signatures of selection, Martinez *et al.* (2011) found three microsatellite markers mapping to Omy5 displaying evidence of a signature of differential selection between two historically sympatric populations of migratory and resident rainbow trout. One of those markers, Omm1009 falls within the 95% confidence interval for the QTL for skin reflectance (AvgPix) here and underlies all the QTL found in Nichols *et al.* (2008) on this chromosome.

In this study, chromosome Omy12 exhibited the largest number of trait associations, including many traits that showed significant phenotypic differences among smolts and nonsmolts. Among those traits was the binary life-history classification (SMOLT), which captures multiple morphological and physiological characteristics between the life-history types as an overall qualitative measure of the life-history tactic of an individual. QTL for body condition factor (Kfact905 and Kfact606), growth rate (IGRL2 and IGRW2), and morphology (RelW3) also map to this chromosome. In Nichols *et al.* (2008), a single QTL for body morphology was identified on this chromosome, which also explained variation in overall head shape and body length. Wringe *et al.* (2010) has identified QTL for body weight and body condition from several crosses of rainbow trout that localize to this chromosome as well, suggesting that conserved genetic mechanisms within this species associated with growth and condition localize to this region. Additionally in a QTL analysis of osmoregulatory capacity in resident rainbow trout, Le Bras *et al.* (2011) identified three QTL on chromosome Omy12 for traits associated with seawater adaptability. Martinez *et al.* (2011) identified a single marker that maps to this chromosome, which shows marginal support of a signature of divergent selection between two closely related populations of rainbow and steelhead trout from California. Given that several smoltification-related traits and the binary life-history trait (SMOLT) colocalize to this position in this cross and additional evidence in other studies suggest loci associated with migration localize to this region (Le Bras *et al.* 2011; Martinez *et al.* 2011; Wringe *et al.* 2010), it is possible that this QTL represents a master genetic switch for the smoltification process in this species.

QTL for morphological variation in body shape have been found between both Nichols *et al.* (2008) and here (RelW3, RelW4, and RelW9) on chromosome Omy14. Although direct comparisons of the components of shape variation between the two studies are not appropriate, components from both studies explain variation in dorsal-ventral body depth and caudal peduncle length, suggesting to some degree a common gene(s) contributing to this shape variation localizes to this region in rainbow trout. We also identified QTL for growth rate (IGRL1 and IGRW1) and body condition factor (Kfact905 and Kfact606) on this chromosome. Wringe *et al.* (2010) have also identified significant QTL for body weight and suggestive QTL for condition factor across multiple families of rainbow trout in this region, suggesting that a conserved genetic mechanism for growth lies within this QTL. The traits IGRW1, Kfact905, Kfact606, and RelW3 were all significantly different between the life-history types and map to the same QTL position on Omy14, suggesting that this locus in addition to the locus on Omy12 plays a substantial role in the smoltification process within this cross.

Among the functional traits of seawater adaptation measured in this study, only a single QTL (on Omy11) for blood plasma sodium concentration (BPNa) after a 24-hr challenge in seawater was found. This QTL represents an important functional physiological response to salt water stress and an individual’s ability to acclimate to seawater (McLeese *et al.* 1994). Le Bras *et al.* (2011) also identified a QTL for plasma sodium ion concentration in this region in addition to QTL for plasma chloride ion concentration and total gill index after seawater challenge. This suggests a conserved functional mechanism for osmoregulatory ability localizes to this region in this species. Furthermore, Martinez *et al.* (2011) found some evidence of differential selection for two microsatellite loci mapping to this chromosome between closely related populations of migratory and resident rainbow trout. And although no additional QTL localize to this position, the evidence here and in other studies suggests the presence of a putative candidate gene(s) in this region that contributes to seawater adaptation during migration.

Nichols *et al.* (2008) identified a single region for which multiple smoltification-related traits mapped in a doubled haploid mapping family of rainbow and steelhead trout on chromosome Omy10, but this same region was not identified for multiple trait associations in our study. If the QTL on chromosome Omy10 truly harbors a conserved master genetic switch for migration *vs.* residency in this species, we should expect that the same region would be responsible for a significant proportion of the variation in migration-related traits in the segregating cross used here. However, we found no evidence of QTL localizing to any position on Omy10. It is possible that a smoltification-associated haplotype in this region is fixed within this population and, therefore, does not segregate. It is also possible that with the F_2_ breeding design used here, in which we relied on within population genetic variation to identify QTL, we didn’t have the power to detect these QTL that Nichols *et al.* (2008) had with their doubled haploid cross from two divergent strains of *O. mykiss*, in which fixed differences between the strains resulted in significantly more power to detect QTL (Lynch and Walsh 1998; Martinez *et al.* 2005). It is also possible that while the QTL may segregate within the Sashin population, it did not segregate in this single QTL mapping family and was, therefore, undetected. And finally, it is possible that different genetic mechanisms for smoltification have evolved between the two mapping populations, where each population was exposed to different selection pressures.

Little is known of the genetic architecture underlying migratory life histories in any species. In large part this lack of understanding stems from a general deficiency of genetic resource or difficulty in quantifying migratory traits in migratory species. Many of the best studied migratory species are birds (Liedvogel *et al.* 2011), and generating segregating experimental crosses in birds can be challenging if not impractical. Although it is possible to perform QTL analyses in wild populations using multigenerational pedigrees (Slate *et al.* 2002; Tarka *et al.* 2010), none that we are aware of have identified QTL for suites of multiple morphological or physiological traits associated with migration *vs.* residency. High-throughput genotyping-by-sequencing technology, including reduced representation library RAD-tag sequencing (Baird *et al.* 2008; Miller *et al.* 2007) used here, is now available to nonmodel species for the development of high-density SNP linkage maps, making gene mapping possible in nonmodel systems (Amores *et al.* 2011). These technologies will in part allow for the expanded investigation of QTL associated with migration-related traits in nonmodel systems (Slate *et al.* 2009) across taxa and allow for between-taxa comparisons of the number, position, and effects of QTL for similar traits.

Across-taxa quantitative genetic methods have long since determined that substantial additive genetic variance is responsible for traits and behaviors associated with migration (Berthold 1988; Dingle 1991; Pulido and Berthold 2003; Pulido *et al.* 2001; Roff and Fairbairn 2007), and we are only beginning to understand the genetic and molecular mechanisms involved in variation in migration tendency. Some studies have identified QTL for variation in single traits associated with migration, such as wing length in birds (Schielzeth *et al.* 2012; Tarka *et al.* 2010), while others have identified candidate genes and hormones associated with migration-related traits in birds and insects (Fidler *et al.* 2007; Mueller *et al.* 2011; Niitepold *et al.* 2009; Zera and Denno 1997). Gene expression studies have identified genes differentially expressed in migratory individuals during periods of residency *vs.* periods of migration, including, for example, studies in white-crowned sparrows (Jones *et al.* 2008), monarch butterflies (Zhu *et al.* 2009), and Atlantic salmon (Seear *et al.* 2010). And comparisons between gene expression studies in closely related salmonid species have even identified the same candidate genes; for example, between Atlantic salmon and brook charr (Boulet *et al.* 2012; Seear *et al.* 2010) and between Atlantic salmon and brown trout (Giger *et al.* 2008). And although some candidate genes have surfaced as being associated with migration-related traits or even the propensity to migrate, it is still unclear how these genes function within the framework of a migratory syndrome.

Here we identify regions highlighted in multiple investigations of the genetic basis of smoltification-related traits (Le Bras *et al.* 2011; Nichols *et al.* 2008) and other adaptive life-history tactics (Miller *et al.* 2012; Nichols *et al.* 2007; O’Malley *et al.* 2003; Robison *et al.* 2001; Wringe *et al.* 2010) within *O. mykiss*. The comparison of these studies suggests some genetic loci are conserved to play a critical role in life-history variation, the smoltification process, and migration. Our results, along with those from Nichols *et al.* (2008), have allowed for a within-species comparison of the genetic architecture of migration from two divergent populations of migratory steelhead trout, and they permit us to develop hypotheses about the evolution of migration within this species. Both studies have identified two QTL regions in particular that have multiple migration-related traits mapping to them; however, the regions are different between the studies. This suggests, in part, that locally adapted gene or gene complexes could play a significant role in the smoltification process within this species. However, given that substantial QTL on Omy5, Omy10, Omy11, and Omy12 have been identified in multiple studies as being associated with life-history variation and migration-related traits in rainbow and steelhead trout, we believe there is some conservation of the genetic mechanisms controlling this process between populations within this species. Our combined results suggest a complex polygenic architecture underlying migration-related traits in this species, as previously hypothesized for migration (Dingle 1991), and provide the foundation for understanding the genetic mechanisms underlying smoltification and migration-related traits within this species and across taxa.

## Supplementary Material

Supporting Information
