# Supplementary material for: Genetic Architecture of Migration-Related Traits in Rainbow and Steelhead Trout, Oncorhynchus mykiss
Source: G3 (Bethesda). 2012 Sep 1;2(9):1113–27. doi: 10.1534/g3.112.003137 (PMC3429926; doi:10.1534/g3.112.003137)
Supplement: Supporting Information [file supp_2_9_1113__index.html]

Supporting Information 

# Genetic Architecture of Migration-Related Traits in Rainbow and Steelhead Trout, *Oncorhynchus mykis*s

## Supporting Information for Hecht *et al.*, 2012

**Files in this Data Supplement:**

- Supporting Information - Files S1-S3 and Tables S1-S5 (PDF, 121 KB)
- File S1 - Supplementary Materials and Methods (PDF, 115 KB)
- File S2 - Supporting Data (.txt, 185 KB)
- File S3 - Supporting Data (.csv, 394 KB)
- Table S1 - Non-RAD-tag based markers in linkage map, the chromosome and position they map to, type of marker, Genbank accession number, forward and reverse primer sequences, and 5' forward primer modification if any (.xlsx, 72 KB)
- Table S2 - A description of the body morphology defined and percent of the variation in total body shape explained by each relative warp (.xlsx, 30 KB)
- Table S3 - Pairwise correlation matrix of 27 smoltification associated phenotypes used in QTL analyses (.xlsx, 59 KB)
- Table S4 - DNA sequences for each allele from 8,789 polymorphic RAD tag loci identified in the Sashin Creek QTL mapping family (.xlsx, 564 KB)
- Table S5 - Genetic map position (cM) and chromosome and linkage group assignment for all markers used to estimate the linkage map (.xlsx, 64 KB)
