# Supplementary material for: Genetic Architecture of Migration-Related Traits in Rainbow and Steelhead Trout, Oncorhynchus mykiss
Source: G3 (Bethesda). 2012 Sep 1;2(9):1113–27. doi: 10.1534/g3.112.003137 (PMC3429926; doi:10.1534/g3.112.003137)
Supplement: Supporting Information [file supp_2.9.1113_003137SI.pdf]

File S1

Supplementary Materials and Methods

Genetic Crosses: Genetic analysis of smoltification related traits were conducted in an F_2 out-crossed family derived from wild migratory steelhead and resident rainbow trout from the Sashin Creek system on Baranof Island in southeast Alaska (56 degrees 23' N, 134 degrees 39' W). In 1926 cannery workers transplanted an unknown number of wild juvenile rainbow trout, with unknown migratory life history, from the lower portion of Sashin Creek above two barrier waterfalls into a previously barren Sashin Lake (Anonymous 1939; Thrower *et al.* 2004a; Thrower *et al.* 2004b). The waterfalls are unsurpassable to upstream migrants, effectively isolating the transplanted population of rainbow trout from the mixed resident and migratory stock from which they were derived. Over the proceeding generations it is purported that selection for residency above the barrier waterfalls would be strong and that those alleles that contribute large genetic effects to a migratory life history would be purged from the gene pool sequestered above the falls. A Cross between resident rainbow trout from Sashin Lake and migratory steelhead trout returning to Sashin Creek would allow for the identification of loci contributing large genetic effects to traits associated with the smoltification process and ultimately migration. In June of 1996 two F_1 families (A_1xR_1 and A_2xR_2) were generated from the gametes of two wild migratory *O. mykiss* females (A_1 and A_2) returning to Sashin Creek, and two wild resident *O. mykiss* males (R_1 and R_2) from Sashin Lake. F_1 progeny were hatched and reared at the Little Port Walter Research Station (Baranof Is., AK) until the age of smoltification (~2 years age) when the binary life-history classification could be qualitatively determined in migratory smolts by an assessment of body morphology and silver coloration. Juvenile smolts were tagged for identification with passive integrated transponder (PIT) tags (Biomark, Boise, ID) and transferred to a marine net pen (30 ppt saltwater) where they were allowed to complete the ocean phase of their life history in captivity. In June of 2004 a mature anadromous F_1 female from family A_1xR_1 was crossed to an unrelated mature anadromous F_1 male from family A_2xR_2. Of the resulting F_2 offspring from this cross, 235 were hatched and reared in similar freshwater conditions as the F_1 and as described further in Thrower *et al.* (2004b).

Genotyping: *Microsatellite Markers* Microsatellite markers were genotyped using either forward primers directly labeled with a 5' fluorescent label (6-FAM, VIC, NED or PET) or forward primers with a 5' M13(-21) tail sequence and fluorescently labeled (6-FAM, VIC, NED, or PET) universal M13(-21) primer in PCR (Schuelke 2000). Microsatellites were pooled into multiplex panels for PCR (up to 4x for individually labeled primers, and 2x for M13 tailed primers)

based on fluorescent label, PCR amplification conditions, and amplicon size. Microsatellites were separated by electrophoresis on the ABI 3130xl (Life Technologies Corporation, Carlsbad, CA), and then scored using Genemapper v4.0 (Life Technologies Corporation, Carlsbad, CA).

The reaction recipe for directly labeled primers included a 5 uL total reaction volume with 2x Type-It Microsatellite PCR Master Mix (Qiagen, Valencia, CA), 0.2 uM each forward primer (no more than four unique forward primers), 0.2 uM each reverse primer (no more than four unique reverse primers), 25 ng DNA, and ddH_2O to volume. PCR reaction conditions consisted of an initial denature step at 95° for 5 min followed by 30 cycles of 95°C for 30 s, 57°C for 45 s, and 72°C for 30 s, with a final elongation step of 60°C for 10 min, and a cool down to 10°C.

The reaction recipe for M13(-21) tailed primers (Schuelke 2000) included a 5 uL total reaction volume with 2x Type-It Microsatellite PCR Master Mix (Qiagen, Valencia, CA), 0.1 uM each forward primer (with no more than two unique forward M13(-21) tailed primers), 0.2 uM each reverse primer (no more than two unique reverse primers), 0.2 uM universal M13(-21) fluorescent labeled primer (no more than one M13(-21) tailed fluorescent label), 25 ng DNA, and ddH_2O to volume. PCR reaction conditions consisted of an initial denature step at 95°C for 5 min followed by 30 cycles of 95°C for 30 s, 57°C for 45 s, and 72°C for 30 s, with a final elongation step of 60°C for 10 min, and a cool down to 10°C.

Microsatellites were multiplexed based on size and fluorescent label, with up to eight markers for electrophoresis scoring on an ABI 3130xL Genetic Analyzer (Life Technologies Corporation, Carlsbad, CA). In preparation for fragment analysis on the 3130xL, PCR products were diluted up to 1:10 in ddH_2O and prepared following the manufacturers recommended protocols (Life Technologies Corporation, Carlsbad, CA). GeneMapper v4.0 (Life Technologies Corporation, Carlsbad, CA) was used to score microsatellite genotypes, and all genotypes were also validated manually by eye.

Sex Marker A genetic sex marker *OmyY1* used herein as a proxy for phenotypic sex was genotyped as described by Brunelli *et al.* (2008). The accuracy of this sex marker within the Sashin creek population was verified in a mixed sample of 190 known-sex, mature resident fish from Sashin Lake and mature migratory fish returning to Sashin Creek. Phenotypic sex was determined by the positive expression of gametes when gentle pressure was applied to the

abdomen of the fish. Of the 190 fish sampled, 101 were phenotypic females and 89 were phenotypic males. The phenotypic sex was compared to the genotype at the putative sex marker OmyY1. PCR reactions that failed to produce an autosomal control product were not considered in the test of accuracy. In total 176 of the 190 samples were successfully genotyped for OmyY1, and 171 of the 176 samples had a genotype that corresponded to the phenotypic sex of the fish, giving a 97% ((171/176)*100 = 97%) accuracy of this marker in the Sashin Creek rainbow and steelhead trout population.

SNP Markers Genotyping and SNP discovery in RAD-tags was performed using perl scripts and a pipeline provided in Miller *et al.* (2012) and the alignment program Novoalign (Novocraft Technologies, Selangor, Malaysia). SNPs were discovered using filtered and sorted reads from two P_1 grandparents, one anadromous steelhead and the other a resident rainbow trout, which had the greatest and most similar sequencing depth of the four (tissue and DNA was limited for the other two grandparents). These two grandparents were used as the sequence index and were overly sequenced compared to F_2 progeny in order to confidently identify as many polymorphic loci as possible. Before calling the genotype of an individual at any given locus, all genotypes were filtered to only include genotypes for a locus if the sum of the two alleles at the locus was greater than or equal to eight, which was the mean read depth for all alleles present across the 119 samples at the 8,790 polymorphic loci. Genotypes were determined by considering the Log_{10} ratio of the two allele counts at a locus ($Log_{10}(A/B)$). Homozygous genotypes were called if the log_{10} ratio of the two alleles were < -0.95, > 0.95, or if the count of one allele was greater than or equal to eight while the other allele was absent. Heterozygous genotypes were called if the log_{10} ratio of the two alleles was between -0.5 and 0.5, which is approximately one standard deviation (0.5) from the mean ratio 0.06 in a distribution of ratios from this dataset (data not shown).

Genetic Linkage Map and QTL Analysis: Genetic linkage map construction was performed using a subset of 119 F_2 individuals that had been genotyped for both microsatellite and RAD-tag SNP markers. Markers were filtered from the analysis if missing more than 30% data, or if any doubled haploid sample was genotyped as a heterozygote (Miller *et al.* 2012). Markers were also removed for exhibiting extreme segregation distortion, determined by using chi-square tests and a Bonferroni corrected p-value ($p = 0.05/659 = 0.000076$). The genetic linkage map was constructed using the R statistical software package 'onemap' (Margarido *et al.* 2007) and the input data file is included as a supplementary file S2. Initially, two-point recombination fractions were estimated between all markers using default

parameters (LOD = 3.0, max. recombination fraction = 0.5). Markers were joined using the 'group' function with a minimum LOD threshold of 8 and maximum recombination fraction of 0.35. Once an initial set of marker groupings were estimated, an additional round of grouping was performed in an attempt to assign ungrouped markers at a LOD of 5 and maximum recombination fraction of 0.5 to the previously grouped markers. Markers that could not be grouped unambiguously at LOD = 5 were discarded from further analysis.

Linkage mapping was performed for each group using the 'order.seq' function with the 'touchdown' option. This function begins map construction with the user defined number of most informative markers within the group in order to generate a framework, after which less informative markers are added in turn at a LOD of 3, until all grouped markers have been mapped. Markers that can be mapped without ambiguity comprise the 'safe' order of markers. Using the 'safe' order, and invoking the 'touchdown' option in 'order.seq' allowed an additional round of mapping with a decreased LOD threshold of 2. Markers that mapped to both telomeres of the linkage group with similar LOD scores were removed from further analysis. If markers were removed, the map was re-estimated using the 'order.seq' function once again. Map order was rippled using a marker window size of five. The best marker order determined by likelihood was used to estimate the final map.

Quantitative trait loci analyses were performed using single, multiple, and two-dimensional QTL models in the R statistical software package 'qtl' (Broman *et al.* 2003). The input file containing all genotypes and phenotypes for import into r/qtl has been provided as a supplementary file S3. Missing genotypes as a result of the selective genotyping scheme used herein were simulated using the 'sim.geno' function with a step size of 2 cM, 128 simulation replicates, and a genotype error probability of 0.01. Genome- and chromosome-wide significance thresholds for multiple QTL models were determined by 1000 permutations of the data (Churchill and Doerge 1994; Doerge and Churchill 1996). To obtain these permutation thresholds given the selective RAD-tag SNP genotyping performed, the dataset was divided into two subsets for stratified permutation testing (Broman and Sen 2009). One stratification contained all individuals genotyped at 176 or more loci, and the other contained those genotyped at fewer than 176 loci. Single QTL were identified using the imputation method, and a normal model for continuous traits, or a binary model for the binary life history classification ("SMOLT") using the 'scanone' function of r/QTL. Some traits were also modeled with sex ("SMOLT", "Weight905", "Kfact905", "Kfact606", "IGRL2", "IGRW2", "AvgPix", "PWL", "BPNa", "RelW2", and "RelW3"), "Centroid_Size" ("RelW4" and "RelW9"), or both sex and "Centoid_Size" ("RelW6") as

additive cofactors. In an effort to detect and explore QTL of both large and small effect, yet keep false positive QTL discovery to a minimum, a significance threshold of LOD ≥ 3 was applied for all traits in single QTL models (Lander and Kruglyak 1995). In every case this threshold was more stringent than a chromosomewide threshold at the α = 0.05 level, but on occasion less stringent than a genomewide threshold at the α = 0.1 level. In the second step, QTL above the established threshold were used as cofactors in a search for additional QTL. Additional QTL found were added to the list of QTL cofactors from the first round, and an additional round of testing was performed. This process was repeated until no new QTL were identified. Two-dimensional QTL models (epistasis) were fitted for traits that had more than one main effect QTL. Significant interactions were fitted to a multiple QTL model with significant main effects. Once a full model of main and interaction effects was established, QTL positions were refined by iteratively testing the likelihood of each QTL position in the context of the full model, until the most likely position of each QTL was determined. Once QTL positions were refined, the full multiple QTL model was fit in order to obtain main and interaction term statistics, as well as the percent variation explained (PVE) by each term of the QTL model. Once this model was fit, if any main effect or interaction was not found to be significant at the chromosomewide α = 0.05 level or greater, it was removed from the full QTL model. If QTL were removed, remaining QTL positions were refined again, and the model was re-fit in the absence of the insignificant QTL until only significant QTL and interactions remained. Two-LOD support intervals, which have been shown to provide conservative estimates of a 95% confidence interval for QTL (Visscher *et al.* 1996) were estimated for each identified QTL. The genetic linkage map and QTL were visualized in the software program MapChart v2.2 (Voorrips 2002).

Literature Cited

Anonymous, 1939 Trout Planting in Alaskan Lakes. The Progressive Fish-Culturist **46:** 31-32.

Broman, K. W., and S. Sen, 2009 *A Guide to QTL Mapping with R/qtl*. Springer, New York.

Broman, K. W., H. Wu, S. Sen and G. A. Churchill, 2003 R/qtl: QTL mapping in experimental crosses. Bioinformatics **19:** 889-890.

Brunelli, J. P., K. J. Wertzler, K. Sundin and G. H. Thorgaard, 2008 Y-specific sequences and polymorphisms in rainbow trout and Chinook salmon. Genome **51:** 739-748.

Churchill, G. A., and R. W. Doerge, 1994 Empirical threshold values for quantitative trait mapping. Genetics **138:** 963-971.

Doerge, R. W., and G. A. Churchill, 1996 Permutation tests for multiple loci affecting a quantitative character. Genetics **142:** 285-294.

Lander, E., and L. Kruglyak, 1995 Genetic dissection of complex traits: guidelines for interpreting and reporting linkage results. Nat Genet **11:** 241-247.

Margarido, G. R., A. P. Souza and A. A. Garcia, 2007 OneMap: software for genetic mapping in outcrossing species. Hereditas **144:** 78-79.

Miller, M. R., J. P. Brunelli, P. A. Wheeler, S. Liu, C. E. Rexroad, 3rd *et al.*, 2012 A conserved haplotype controls parallel adaptation in geographically distant salmonid populations. Molecular Ecology **21:** 237-249.

Schuelke, M., 2000 An economic method for the fluorescent labeling of PCR fragments. Nature Biotechnology **18:** 233-234.

Thrower, F., C. Guthrie, J. Nielsen and J. Joyce, 2004a A comparison of genetic variation between an anadromous steelhead, Oncorhynchus mykiss, population and seven derived populations sequestered in freshwater for 70 years. Environmental Biology of Fishes **69:** 111-125.

Thrower, F. P., J. J. Hard and J. E. Joyce, 2004b Genetic architecture of growth and early life-history transitions in anadromous and derived freshwater populations of steelhead. Journal of Fish Biology **65:** 286-307.

Visscher, P. M., R. Thompson and C. S. Haley, 1996 Confidence intervals in QTL mapping by bootstrapping. Genetics **143:** 1013-1020.

Voorrips, R. E., 2002 MapChart: Software for the graphical presentation of linkage maps and QTLs. Journal of Heredity **93:** 77-78.

Files S2 and S3

Supporting Data

Available for download at http://www.g3journal.org/lookup/suppl/doi:10.1534/g3.112.003137/-/DC1.

Tables S1-S5

Supporting Tables

Available for download at http://www.g3journal.org/lookup/suppl/doi:10.1534/g3.112.003137/-/DC1.

Table S1 Non-RAD-tag based markers in linkage map, the chromosome and position they map to, type of marker, Genbank accession number, forward and reverse primer sequences, and 5' forward primer modification if any.

Table S2 A description of the body morphology defined and percent of the variation in total body shape explained by each relative warp.

Table S3 Pairwise correlation matrix of 27 smoltification associated phenotypes used in QTL analyses. Phenotypic abbreviations are described in the text and in Table 1. Significant correlations are in bold. For each pair of traits, correlation coefficients, P-values for tests of HO : P = 0, and sample size is shown. Italicized pairs are traits for which QTL colocalize.
* Spearman's correlation coefficients; all other correlation coefficients are Pearson's correlation coefficients.

Table S4 DNA sequences for each allele from 8,789 polymorphic RAD tag loci identified in the Sashin Creek QTL mapping family. Allele sequences were aligned against and named according to previously identified RAD tag loci in rainbow trout (R00015 - R40649) (Miller et al. 2011). Unique loci (R40650 - R45621) were assigned a name following the sequential order established in Miller et al. (2011). Allele sequences begin with an eight base sbfI restriction enzyme recognition site and follow with 60 bp of nucleotide sequence.

Table S5 Genetic map position (cM) and chromosome and linkage group assignment for all markers used to estimate the linkage map.
